# Analyzing patient experiences using natural language processing: development and validation of the artificial intelligence patient reported experience measure (AI-PREM)

**DOI:** 10.1186/s12911-022-01923-5

**Published:** 2022-07-15

**Authors:** Marieke M. van Buchem, Olaf M. Neve, Ilse M. J. Kant, Ewout W. Steyerberg, Hileen Boosman, Erik F. Hensen

**Affiliations:** 1 Information Technology & Digital Innovation Department, Leiden University Medical Center, Leiden, the Netherlands; 2 Department of Biomedical Data Sciences, Leiden University Medical Center, Leiden, the Netherlands; 3 Clinical Artificial Intelligence Implementation and Research Lab (CAIRELab), Leiden University Medical Center, Leiden, the Netherlands; 4 Department of Otorhinolaryngology and Head and Neck Surgery, Leiden University Medical Center, Leiden, the Netherlands; 5 Morgens, Leiden, the Netherlands

**Keywords:** Natural language processing, Sentiment analysis, Unsupervised machine learning, Patient satisfaction, Patient-centered care

## Abstract

**Background:**

Evaluating patients’ experiences is essential when incorporating the patients’ perspective in improving healthcare. Experiences are mainly collected using closed-ended questions, although the value of open-ended questions is widely recognized. Natural language processing (NLP) can automate the analysis of open-ended questions for an efficient approach to patient-centeredness.

**Methods:**

We developed the Artificial Intelligence Patient-Reported Experience Measures (AI-PREM) tool, consisting of a new, open-ended questionnaire, an NLP pipeline to analyze the answers using sentiment analysis and topic modeling, and a visualization to guide physicians through the results. The questionnaire and NLP pipeline were iteratively developed and validated in a clinical context.

**Results:**

The final AI-PREM consisted of five open-ended questions about the provided information, personal approach, collaboration between healthcare professionals, organization of care, and other experiences. The AI-PREM was sent to 867 vestibular schwannoma patients, 534 of which responded. The sentiment analysis model attained an F1 score of 0.97 for positive texts and 0.63 for negative texts. There was a 90% overlap between automatically and manually extracted topics. The visualization was hierarchically structured into three stages: the sentiment per question, the topics per sentiment and question, and the original patient responses per topic.

**Conclusions:**

The AI-PREM tool is a comprehensive method that combines a validated, open-ended questionnaire with a well-performing NLP pipeline and visualization. Thematically organizing and quantifying patient feedback reduces the time invested by healthcare professionals to evaluate and prioritize patient experiences without being confined to the limited answer options of closed-ended questions.

**Supplementary Information:**

The online version contains supplementary material available at 10.1186/s12911-022-01923-5.

## Background

Patient-centeredness is an essential fundament for providing high-quality care [1, 2]. Insight into the patient-centeredness of care is obtained by evaluating patient experiences, typically using Patient-Reported Experience Measures (PREMs). Most PREMs include a combination of closed- and open-ended questions. When presented with both, healthcare professionals tend to value the answers to open-ended questions most [3]. These answers can be used to identify new points of interest (‘topics’) and provide context to closed-ended questions [3, 4]. Although the value of open-ended questions is widely recognized, patients’ free-text answers remain underutilized in clinical practice. One of the key challenges lies in the time needed for analysis. The answers to open-ended questions are often manually analyzed, which is laborious and time-consuming [3], especially in larger groups of patients.

Several studies aim to automate the analysis of free-text patient experience data to inform quality improvements, showing promising results [5–15]. Most of these studies concentrate on publicly available social media or forum data, usually focused on reviewing hospitals or physicians [5–9]. Current approaches include the use of artificial intelligence (AI) methods such as machine learning and natural language processing (NLP). A few studies successfully applied NLP techniques to routinely collected PREM questionnaires of patients [10–15]. Most of these studies use supervised methods; for example, topic classification is used to classify data into predefined, manually extracted topics [5, 7, 11, 13, 15]. Although some of these methods perform well, supervised methods lack the capability of finding new or unexpected topics. Moreover, regular manual labeling is time-consuming and, therefore, not suited to decrease the current burden of reading through the patients’ answers [10]. Using unsupervised methods such as topic modeling can overcome these limitations. Two studies have compared supervised topic classification to unsupervised topic modeling and concluded that topic modeling leads to topics similar in quality [7, 15].

Current open-ended questions are often unsuitable for automatic analysis as they were not developed for this purpose [10, 11]. An example is a questionnaire consisting of the questions ‘What did we do well?’ and ‘What could we improve?’. Previous work shows that answers to both questions can be positive and negative, complicating automated sentiment analysis [10, 11]. One study created a new, open-ended questionnaire suitable for analysis with NLP [16], focusing on patient-reported outcomes instead of experiences. They concluded that adding open-ended questions leads to richer, more in-depth information, and analysis with NLP makes it feasible to use in clinical practice.

The aim of this study is to harness the value of free-text patient experiences, using NLP methods that have the flexibility to find new topics in a complex, fast-changing environment. Our approach is to develop and validate a method for collecting and analyzing open-ended PREMs that could be incorporated into clinical practice. This objective contains three sub-objectives:

1. Develop and validate an open-ended generic PREM questionnaire;
2. Develop and validate an NLP pipeline to automatically analyze the open-ended PREM;
3. Develop a visualization that supports healthcare professionals in identifying quality improvements from the results.

## Methods

**Fig. 1:**
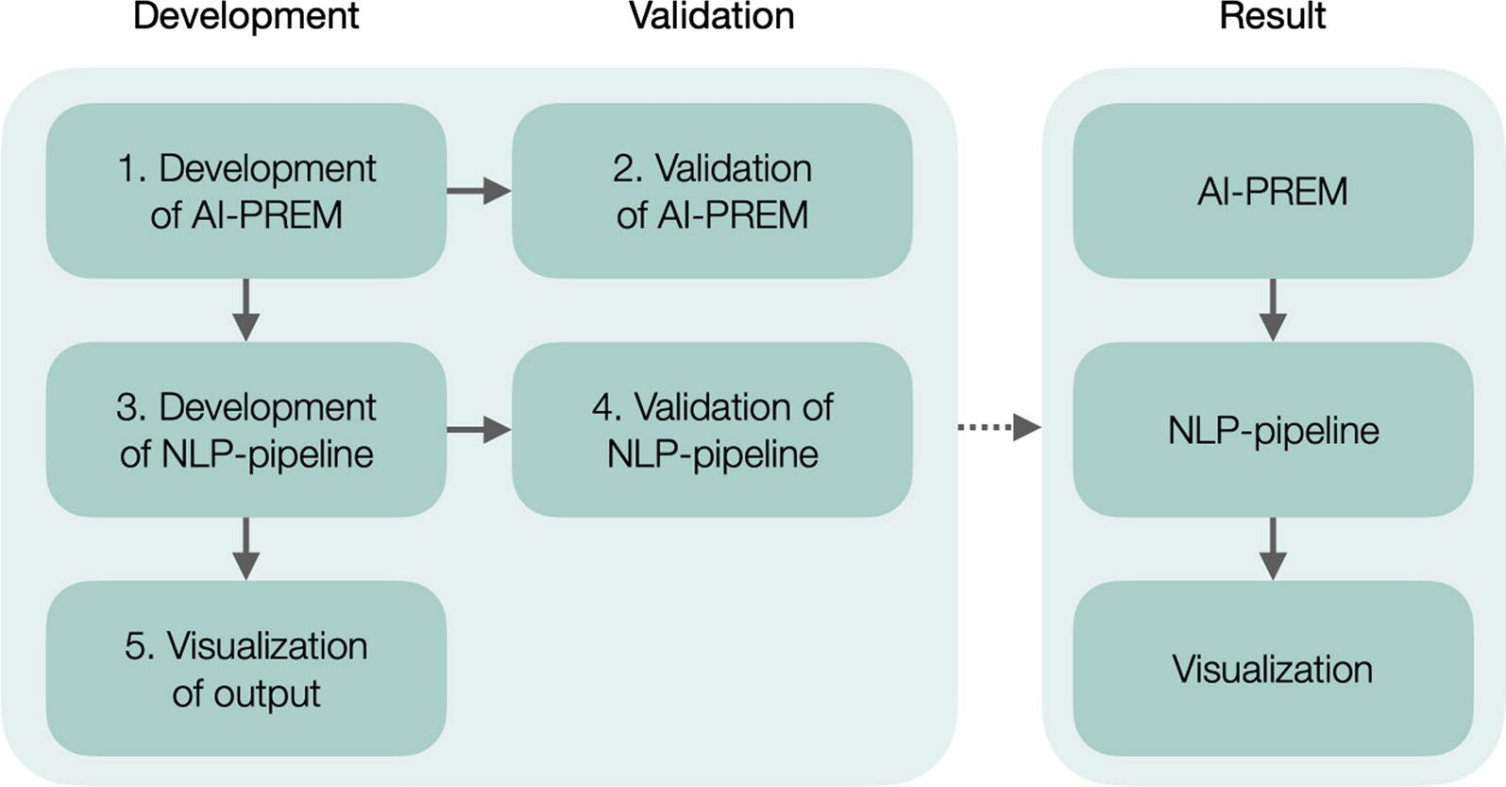
Overview of the different tasks and phases

We devised a method that included a new, open-ended questionnaire, an NLP pipeline to analyze the questionnaire, and a visualization of the output of the NLP pipeline (Fig. 1). This project was organized in a development phase and a validation phase. The development phase started with developing a new questionnaire, the Artificial Intelligence Patient-Reported Experience Measure (AI-PREM).

### Development of the AI-PREM (Fig. 1, step 1)

The AI-PREM was developed iteratively with patients from the vestibular schwannoma care pathway in the Leiden University Medical Center (LUMC) (Box 1). We used the following criteria: (1) Open-ended questions; (2) Phrasing suitable for analysis with NLP; (3) Generic questions, therefore not containing disease-, department-, or center-specific questions; (4) Accessible in terms of length and language. The Picker principles of patient-centered care [17] were the basis for the questionnaire. The development process started with questions about all eight Picker principles, asking patients about experiences with the accessibility of care, continuity of care, involvement of family, emotional support, information provision, physical needs, and involvement in decisions. Each question included one subject and did not contain a sentiment, to decrease the variability of patients’ answers. For example, instead of asking ‘What could be improved in the organization of care?’ the question stated ‘How was the organization of care?’. These questions were evaluated and finetuned in a group of patients.

**Box 1:** Description of the vestibular schwannoma care pathway in the LUMC

Vestibular schwannomas are benign intracranial tumors, with a heterogeneous clinical presentation: it may present as a small, slow growing, and asymptomatic tumor, but also as large, faster growing, and potentially fatal disease. Patients typically present with symptoms of hearing loss, loss of balance and vertigo, but may also suffer from facial numbness, facial paralysis, or elevated intracranial pressure. In non-progressive tumors, active surveillance with MRI is usually the management option of choice. In progressive tumors, surgery or radiotherapy is performed to prevent future complications. After an active intervention, prolonged active surveillance ensues in these patients too, in order to identify possible recurrences. The LUMC is an expert referral center for vestibular schwannoma in the Netherlands. The care is organized in an integrated practice unit including all specialties involved in the diagnosis and treatment (i.e., neurosurgery, otorhinolaryngology, radiology and radiation oncology).	

Patients who participated in a survey study in 2014 were re-approached for participation in the AI-PREM project between May and September 2020 [18]. Patients that agreed to participate provided their written informed consent. All patients were diagnosed with unilateral vestibular schwannoma between 2003 and 2014. Patients with bilateral vestibular schwannoma, other skull base pathologies, or insufficient proficiency in the Dutch language to complete the questionnaires were excluded. In addition to the AI-PREM, patients were also asked to fill out a validated structured patient experience questionnaire, the patient experience monitor (PEM), for comparison [1]. Patients first filled out the AI-PREM to ensure they were not biased towards the topics assessed in the PEM. The questionnaires were sent out either by e-mail using Castor software or hard copy by mail. These hard copies were verbatim digitalized manually.

### Validation of the AI-PREM (Fig. 1, step 2)

To validate the AI-PREM questionnaire, we used the COSMIN reporting guideline for studies on the measurement properties of patient-reported outcome measures [19]. Although this guideline is aimed at structured questionnaires about patient outcomes, most parts can be applied to unstructured patient experience questionnaires. The COSMIN guideline investigates the content validity of questionnaires by looking at the questions’ relevance, comprehensiveness, and comprehensibility. We examined the content validity of the AI-PREM by comparing AI-PREM questions to similar questions from the PEM. First, a sentiment analysis (as described in the *Sentiment analysis* section under ‘Development of the NLP pipeline’) was performed, labeling a text as positive or negative feedback. We hypothesized that patients who were negative about certain aspects of care in the AI-PREM would also give lower scores on the matched PEM questions and vice versa (scores range from one to ten, where one is the lowest and ten is the highest). Therefore, we defined ‘positive’ and ‘negative’ comments per AI-PREM question based on the sentiment analysis. Per AI-PREM question, we took the matched PEM questions and calculated the average score for the ‘positive’ and ‘negative’ groups. Using a t-test for independent samples, we compared the average scores between the ‘positive’ and ‘negative’ groups.

### Development of the NLP pipeline (Fig. 1, step 3)

The pipeline as described by Cammel et al. was taken as a starting point [10]. The pipeline includes sentiment analysis, preprocessing, and topic modeling. We combine a supervised (sentiment analysis) and unsupervised (topic modeling) approach. We use a supervised approach for the sentiment analysis because the categories for this task (positive, neutral, negative) will not change over time, in contrast to the topics that patients mention. The pipeline was developed in an iterative process by a team of data scientists, researchers, and clinicians of the vestibular schwannoma IPU, to fulfill the following pre-set requirements:

- Interpretable: The end-user should be able to distill from the output what patients experience as positive and negative.
- Actionable: The output should be specific enough to lead to concrete action points.
- Complete: The number of texts that cannot be assigned to a topic should be as small as possible.

Once the output met all the requirements according to the development team, the validation phase started.

#### Sentiment analysis

We finetuned a pretrained, multilingual BERT model for two binary classification tasks for sentiment analysis. The first binary classification task classified answers as negative or non-negative; the second task classified the non-negative answers as positive or neutral. To train these two sentiment analysis models, one annotator (MvB) manually labeled 75% of the collected data as ‘negative’, ‘positive’, or ‘neutral’. A second annotator (ON) labeled 1/3rd of this data (25% of the collected data), which was used to calculate the inter-annotator agreement (percentage of datapoints that the annotators agreed on). Annotators labeled an answer as ‘negative’ if it described a topic or situation that the patient was dissatisfied with (e.g., ‘I had to wait for a long time’). If a non-negative answer described a topic or situation that the patient was satisfied with, it was labeled as ‘positive’ (e.g., ‘the personnel was very friendly’). All answers that described a topic or situation that was neither positive nor negative were labeled as ‘neutral’ (e.g., ‘first I was treated at hospital number 1, then I was referred to hospital number 2’). The two sentiment analysis models were trained on a random sample of 80% and validated on the other 20% of labeled data, using the default parameters of the Transformers implementation of the BERT model for Sequence Classification [20].

#### Preprocessing

After the sentiment analysis, the data were preprocessed. We tokenized words and corrected the spelling using the Peter Norvig algorithm [21] and the CyHunSpell Python package [22]. Subsequently, words were lemmatized, and all non-informative words (stopwords, words with less than three letters, and all words except verbs, adverbs, nouns, and adjectives) were removed using the Stanza Python package [23]. Finally, all n-grams ranging from one to three were vectorized using term frequency-inverse document frequency (TF-IDF).

#### Topic modeling

We used topic modeling, specifically Non-negative Matrix Factorization (NMF), to identify the most important topics from the patients’ answers to the AI-PREM, as described by Cammel et al. [10]. NMF was chosen over Latent Dirichlet Allocation because patients’ answers tend to be very short and NMF is better able to deal with short answers. A separate topic model was created per sentiment (positive or negative) and per question. For each topic model, the optimal number of topics was chosen by creating several topic models with topics ranging from 2 to 15 and calculating the coherence score within every topic. The coherence score was calculated using the semantic similarity of words within a topic, based on a Dutch Word2Vec model [24–26], to account for exact matches and synonymous words. The topic model with the highest coherence metric was chosen as the best fitting model for that specific category.

### Validation of the NLP pipeline (Fig. 1, step 4)

We performed different validation steps to evaluate the performance of the NLP pipeline. (1) We assessed whether the automatically defined topics were representative of the texts they described. (2) We evaluated whether the NLP pipeline extracted topics similar to human-extracted topics.

#### Representativeness of the data

We randomly sampled the answers to the AI-PREM and performed manual evaluations of these answers by clinical experts. One clinician (ON) assessed a sample of the texts within the different categories (e.g., positive answers about information, negative answers about the organization of care). Per category, 20% of the answers per topic were analyzed, with a minimum of 10 texts. Some topics included less than ten texts; the clinician evaluated all texts for these topics. For every text within the sample, the clinician decided if it fit within the assigned topic. This analysis resulted in a percentage showing how representative the different topics were for the answers within that topic. A researcher (MvB) went through the same validation process to calculate the inter-annotator agreement.

#### Topic model versus human comparison

To investigate the performance of the topic model compared to human analysis, two clinical experts (a physician and a nurse practitioner) from the vestibular schwannoma care pathway read the answers to the AI-PREM from a sample of 50 patients, as data saturation was reached. A qualitative approach was used to identify topics within these texts. After reading, the experts decided on a few topics per question that summarized patients’ answers in a consensus meeting. Two researchers (MvB and ON) compared these manually selected topics to the automatically selected topics from the NLP pipeline. Because the human analysis consisted of a sample of 50 questionnaires (and not all), we did not try to match exact words but matched on topic level. The proportion of manually identified topics that could be matched to an automatically identified topic was subsequently calculated.

### Visualization of the output (Fig. 1, step 5)

To stimulate the use of the AI-PREM tool in clinical practice, we co-created a mock-up of a potential visualization. We held three feedback sessions with a group of physicians, nurse practitioners, and implementation managers and iteratively updated the visualization based on their feedback and pre-set requirements. The requirements for the visualization were:

1. Applicability within the end-users current workflow;
2. Presentation of an overview of the output at a glance;
3. Ability to get more context without going through all the individual questionnaires.

## Results

### Development of the AI-PREM

During six iterations, the initial questions were finetuned. The most significant changes made during these iterations were reducing the number of questions and simplifying the sometimes abstract Picker principles. The comprehensibility improved by using only level B1 words of the Common European Framework of Reference for Languages [27]. Furthermore, patients preferred to have some examples of what was meant by the different aspects. The Picker institute provides some examples, which we added to each question. This led to the following questions:

- Q1: How was the provided information? Think of: the prognosis, possible tests, and treatment(s)
- Q2: How was the personal approach? Think of: shared decision making, listening to your preferences, emotional support
- Q3: How was the collaboration between healthcare professionals? Think of: no varying advice or having to tell your story multiple times, contact with your family doctor or other hospitals
- Q4: How was the organization of care? Think of: making appointments, combining appointments on one day, availability by phone
- Q5: What else would you like to share about your experience?

In total, 536 out of 867 vestibular schwannoma patients filled out the AI-PREM and PEM questionnaires, resulting in a response rate of 62%. Two patients were excluded because their diagnosis changed from vestibular schwannoma to meningioma, requiring treatment in another care pathway. This resulted in 534 sets of questionnaires. The median length of patients’ answers was two words, with an interquartile range of 1 to 11 words. The maximum length was 192 words.

### Validation of the AI-PREM

Using the Picker principles as a basis, the AI-PREM adhered to the relevance and comprehensibility criteria from the COSMIN reporting guideline. The comprehensibility criterium was further substantiated by including patients in the development of the AI-PREM. The results of validating the last criterium, comprehensiveness, are shown in Table 1. Where Q1-3 showed a significant difference in PEM scores between positive and negative answers, Q4 did not. No PEM questions were matched to Q5 (‘What else would you like to share about your experience?’), so we did not validate this question.

**Table 1:** Overview of the number of AI-PREM responses per sentiment

Questions	Number of patients N (%)	Average PEM scores of matched questions, ranging from 1 to 10 µ ± sd
Q1–Positive Negative	359 (67.2%) 26 (4.9%)	9.7 ± 0.9 8.1 ± 2.4**
Q2–Positive Negative	360 (67.4%) 31 (5.8%)	9.7 ± 0.7 7.7 ± 2.6**
Q3–Positive Negative	325 (60.9%) 40 (7.5%)	9.6 ± 1.1 8.3 ± 1.8*
Q4–Positive Negative	343 (64.2%) 39 (7.3%)	6.9 ± 1.7 6.4 ± 2.0
Q5–Positive Negative	121 (22.7%) 35 (6.6%)	

The neutral responses are left out. Per category (question and sentiment), the average scores to the PEM questions that matched the AI-PREM questions are shown. P-value for the t-test for independent samples: * = *p* < 0.001, ** = *p* < 0.0001. AI-PREM: artificial intelligence patient reported experience measure. PEM: patient experience monitor. Q: question. sd: standard deviation

### Development of the NLP pipeline

**Fig. 2:**
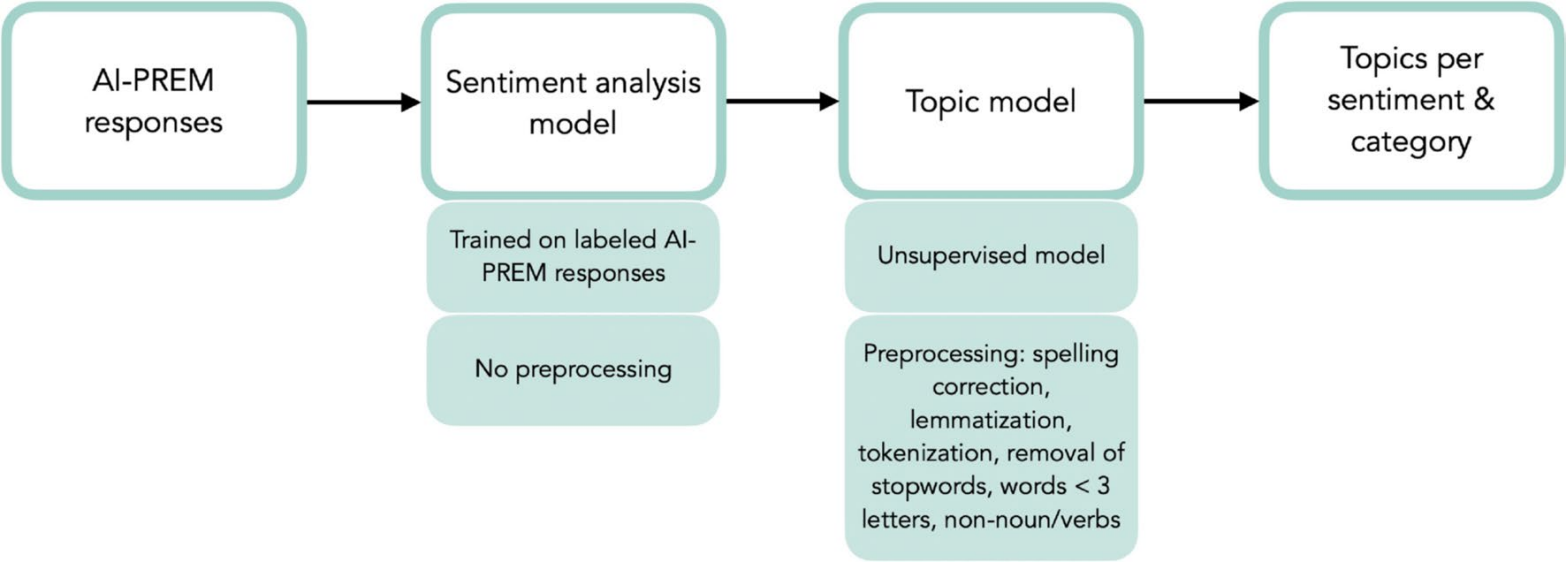
Overview of the input, models, and output of the AI-PREM tool

We made several improvements to the pipeline during the iterative development process (Box 2). The final NLP pipeline contained a sentiment analysis model consisting of a negative and positive sentiment classifier and a topic modeling module (Fig. 2).

**Box 2:** Most important improvements that were made during the iterative development process

– To first perform a sentiment analysis and then create a separate topic model per sentiment and per question, instead of creating one topic model for both sentiments. This led to more specific topics, from which points of improvements could be derived more easily, increasing the interpretability and actionability
– To not only include the negative feedback topics but also the positive ones, in order to obtain more balanced information. This was found to be essential in selecting and prioritizing points of improvement. In addition, the positive topics were seen as motivators for the healthcare team
– To go from a fixed number of topics to an adaptive approach that automatically chooses the optimal number of topics per subject. This increased the completeness
– To add a quantitative dimension to the qualitative output of the topic model, in order to help prioritize aspects of care that need the most attention
– To include n-grams up to three instead of just using 1 g. This increased the interpretability and actionability of the topics

#### Sentiment analysis

The inter-annotator agreement was 91.9%. The precision and recall for the negative sentiment model were 0.78 and 0.53, respectively, with an F1 score of 0.63. The precision, recall, and F1 score for the positive sentiment model were all 0.97.

#### Topic modeling

**Fig. 3:**
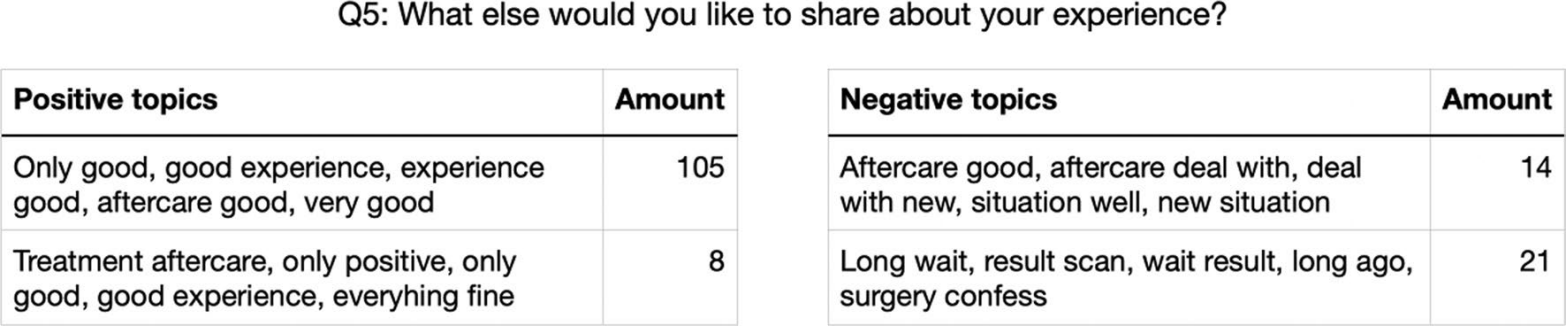
Topic model for Q5

The number of topics per category ranged from two to six. 2.8% of texts could not be assigned to a topic. Only the ten n-grams with the highest TF-IDF score per topic were extracted to increase the interpretability of the topics. These n-grams were sorted based on the number of words, with the highest number of words shown first. We deduplicated this list of words to ensure that the final list of descriptors would not contain both ‘went very well’ and ‘went well’. Finally, the first five words of this sorted, deduplicated list were shown to the end-user (Fig. 3). See Additional file 1 for all the topics per category.

### Validation of the NLP pipeline

The overall percentage of representative texts was 80.9%, with 90.1% for the positive texts and 72.0% for the negative texts (Table 2). The inter-annotator agreement was 94.4% for positive texts, 80.5% for negative ones, and 90.4% overall. The clinical experts extracted 20 topics: 14 for the positive and 6 for the negative texts. All negative topics and 12 of 14 positive topics could be matched to the automatically extracted topics, leading to a 90% overlap between human topics and automatically extracted topics .

**Table 2:** Representativeness of the different topic models per category

Question	Positive categories in total	Per topic	Negative categories in total	Per topic
Q1	94.4% (n = 72)	T1: 100% (n = 36) T2: 88.9% (n = 36)	55.6% (n = 18)	T1: 60% (n = 10) T2: 50% (n = 8)
Q2	93.3% (n = 75)	T1: 97.1% (n = 35) T2: 100% (n = 10) T3: 85% (n = 20) T4: 90% (n = 10)	71% (n = 31)	T1: 100% (n = 3) T2: 100% (n = 3) T3: 83.3% (n = 6) T4: 100% (n = 3) T5: 75% (n = 4) T6: 28.6% (n = 7) T7: 60% (n = 5)
Q3	98.4% (n = 63)	T1: 100% (n = 43) T2: 95% (n = 20)	76.9% (n = 39)	T1: 100% (n = 4) T2: 33.3% (n = 3) T3: 85.7% (n = 7) T4: 100% (n = 5) T5: 66.7% (n = 3) T6: 77.8% (n = 9) T7: 62.5% (n = 8)
Q4	100% (n = 65)	T1: 100% (n = 41) T2: 100% (n = 12) T3: 100% (n = 12)	86.7% (n = 15)	T1: 100% (n = 5) T2: 80% (n = 10)
Q5	86.2% (n = 29)	T1: 85.7% (n = 21) T2: 87.5% (n = 8)	55.5% (n = 20)	T1: 50% (n = 10) T2: 60% (n = 10)

Representativeness is defined as the number of texts within a certain topic that fit the description of the topic. The percentage is calculated by dividing the texts that fit the description of the topic by the total number of texts within the topic. Q: AI-PREM question. T: automatically extracted topic

### Visualization of the output

**Fig. 4:**
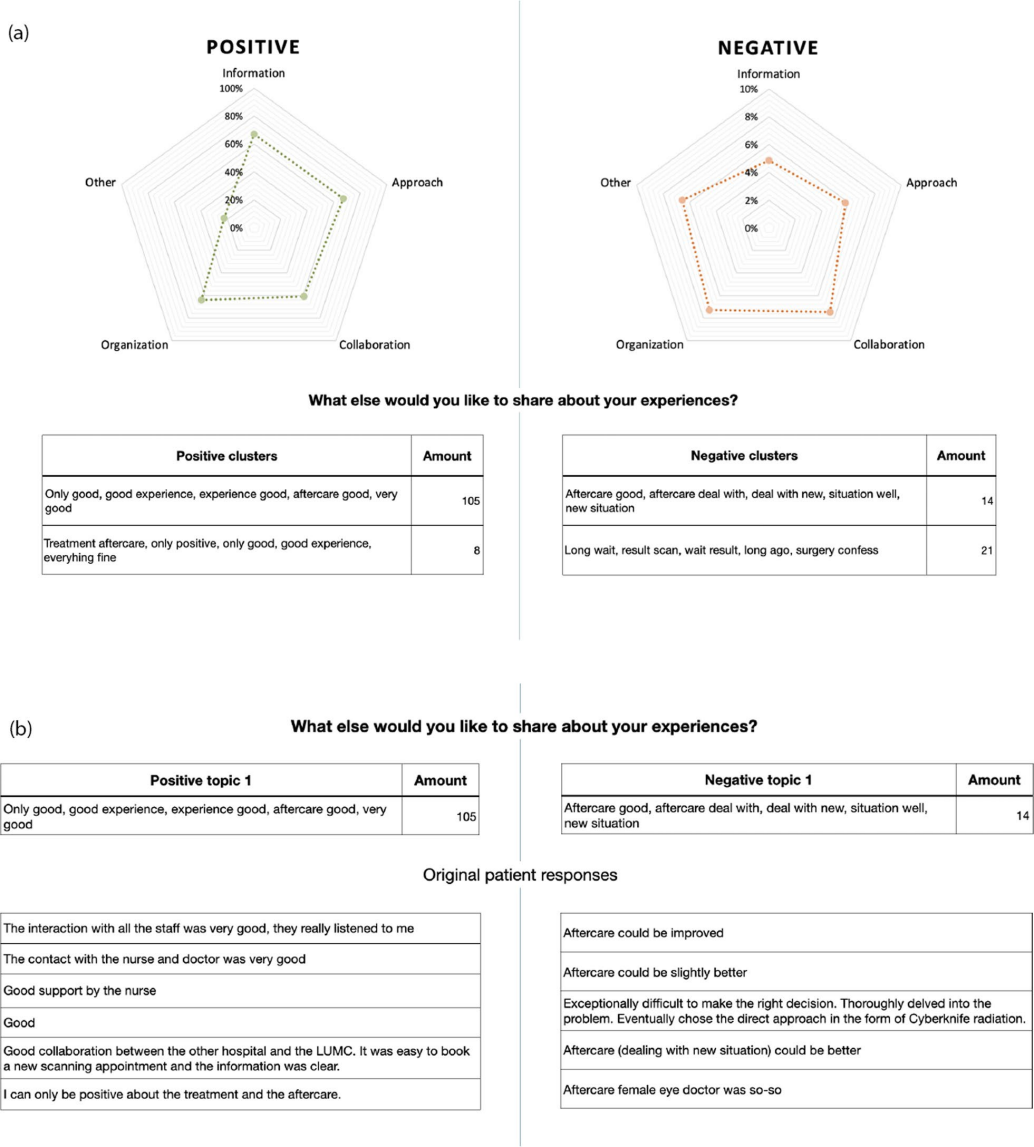
**a** Stage 1: the spider plot showing the percentage of positive and negative texts per question. Stage 2: once the end-user clicks on one of the questions, the automatically extracted topics are shown. The positive topics are shown on the left and the negative topics on the right. **b** Stage 3: if the end-user wants to dive into one of the topics, they can click on that topic and read the actual patient answers that belong to that topic. In this example, the end-user is looking at the topics within the ‘Other’ category and has clicked on positive topic 1 and negative topic 1

The end-users preferred the spider plot over other visualizations in the feedback session, such as a bar plot or tornado graph. The final visualization included a mock-up with three stages (Fig. 4).

## Discussion

This study describes the development and validation of a comprehensive tool for surveying the patient experience that can automatically produce actionable information. The tool consists of an open-ended, validated patient experience questionnaire suitable for qualitative and quantitative analysis with natural language processing (NLP), a well-performing NLP pipeline to analyze the answers to the questionnaire automatically, and a visualization that supports healthcare professionals in defining quality improvements from the results.

A critical aspect of our study is that we created and validated a new questionnaire consisting of only open-ended questions. One other study developed a new, open-ended questionnaire suitable for analysis with NLP, but they focused on patient outcomes instead of experiences [16]. Unique in our study is that we compared the AI-PREM with a ‘gold standard’ PREM, the patient experience monitor (PEM). Overall, three out of four open-ended questions of the AI-PREM seem to capture sentiments similar to the PEM. The lack of a significant correlation for the fourth question, asking about the organization of care, might be explained because this question had the lowest average PEM score and the smallest range.

Our NLP pipeline combines sentiment analysis with topic modeling while also making it possible to go back to individual patients’ original responses per topic. This hierarchical structure allows healthcare professionals to scan the sentiment analysis for a high-level view or dive into the different topics and texts to define quality improvements. Physicians can use the quantitative data to review the results at a glance and prioritize the various topics, while the qualitative data allows them to put the topics into context and define concrete points of action.

Unlike most studies [5, 7, 11, 13, 15], we chose an unsupervised topic modeling approach due to its flexibility in finding new and unexpected topics [3, 10]. One example that highlights the benefit of this approach is the topic describing the negative sentiment patients had about how long they had to wait for the scan results. This topic is not included in structured questionnaires and is very specific to this care pathway. Furthermore, the differing number of topics per question shows the ability of this method to adapt to the data at hand. Methods sensitive to changing topics in patients’ experiences are essential in the constantly changing healthcare environment.

We finetuned a pretrained multilingual BERT model on our data for the current sentiment analysis. Because the questionnaire and answers were in the Dutch language, there was limited choice in off-the-shelf sentiment analysis models, and the available models did not perform well on our data. Furthermore, there are no BERT models pretrained on clinical data for Dutch, so we used the multilingual BERT model as a basis. The positive sentiment model performs better than most other studies, with an F1 score of 0.97. Other studies report F1 scores between 0.74 and 0.90 for sentiment analysis on patient experience data [6, 14, 15, 28, 29]. The negative sentiment model performs below average, with an F1 score of 0.63. The small number of negative texts compared to the amount of neutral and positive texts causes this difference. With more data, the model can be trained further to improve the performance in recognizing negative texts and make it more generalizable to other departments and care pathways.

Our manual validation of the NLP pipeline shows that the quality of the topics is high in terms of the representativeness of the topics and the similarity to the manual topics. These results align with previous studies that show the similarity between supervised, manually defined topics and unsupervised, automatically defined topics [7, 15]. However, there is a large difference in the quality of the topics for the different categories in the AI-PREM. Although most topics represent their texts very well with scores ranging from 90 to 100%, a few mostly negative topics have scores between 20 and 50%. One possible explanation is the heterogeneity in the negative answers, leading to a few ‘left-over’ topics that fail to represent the texts well. One solution would be to gather more data before running the model, as this would decrease the chance of getting topics that only contain a few texts. Another solution is changing the phrasing of the questionnaire by making it more specific or giving different examples. Especially the question about the organization could be improved because this question also showed low responsiveness to changes in sentiment. On the other hand, the number of texts that could not be assigned a topic was only 2.8%, which is much better than the 15.4% reported in previous work [10]. It shows that a larger amount of texts can be automatically analyzed and confirms the improved suitability of our proposed open-ended questions for NLP analysis. In a previous report by Spasíc et al. [16], the authors optimize their questionnaire comprising open-ended questions in a similar way, i.e., by focusing every question on one particular aspect (different patient outcomes in their case), extracting any sentiment from the question itself, and providing examples per question (also at their patients’ request).

We noted that positive comments are much more numerous, but negative topics tend to be more elaborately discussed by patients. For example, the negative topics’ wait result scan’ and ‘contact (with) other hospital’ contain concrete problems, while ‘information good’ and ‘only positive’ are much more high-level. These results align with other studies [3, 11, 30], which also found more specific feedback in negative comments. As we aimed to facilitate the quality improvement process, we see no limitation in this finding: the in-depth nature of the negative feedback makes it possible to define specific points of improvement, while the more general positive feedback functions as motivation for healthcare professionals. Moreover, previous work on structured patient experience questionnaires describes the problem of the ceiling effect: patient experience questionnaires tend to overestimate patient satisfaction [4], and very satisfied patients often still include a point of improvement [5, 31]. The AI-PREM shows this same trend towards positive responses, but the ability to provide a free text response leads to more in-depth feedback. The tool further facilitates healthcare professionals to put topics into perspective by comparing positive to negative topics and forming concrete action points by going back to patients’ original responses.

### Strengths & limitations

A strength is the combination of quantitative data from the sentiment analysis and qualitative data from the topic models, which creates a clear, usable overview of patients’ experiences. It also aligns with the proposed framework for automated analysis of opinionated data from a recent study [32]. This framework presents a similar pipeline, with sentiment analysis for the quantitative analysis followed by a more qualitative approach using, for example, topic modeling.

Another strength of the current study is the validation steps we took to assess the performance of the AI-PREM tool. Although it was challenging to find suitable validation methods, the current methods combined with the COSMIN reporting guideline provide some insight into how well the topics represent the patients’ answers. However, the combination of the small sample size per topic and lack of easily interpretable metrics limits the use of topic modeling. Therefore, we could not compare our topic models to other literature.

The current sentiment analysis model, which assigns a whole text as either ‘positive’, ‘neutral’, or ‘negative’, is limited. By assigning texts as ‘negative’ if they contained at least one aspect that the patient was negative about, we made sure not to miss any points for improvement. However, in the future, we would like to finetune the model to define a sentiment per sentence instead of per text and to change the sentiment into a 5-point scale ranging from ‘very dissatisfied’ to ‘very satisfied’. This granularity would make it easier to define priorities based on the level of dissatisfaction with a specific aspect of care.

Lastly, our current tool was built and validated in close consultation with clinicians, which ensures the internal validity of the model and clinically relevant and actionable output. However, it was validated using the patient experiences of a specific patient group. To investigate the generalizability of the AI-PREM tool, we will have to collect AI-PREM data in other patient groups and evaluate its usability for different groups of physicians.

## Conclusions

The AI-PREM tool is a comprehensive method that combines a validated questionnaire consisting of open-ended questions with a well-performing NLP pipeline and visualization. By thematically organizing and quantifying patient feedback, it reduces the time invested by healthcare professionals to evaluate and prioritize patient experiences without being confined to the limited answer options of closed-ended questions.

## Supplementary Information

**Additional file 1**. Complete overview of the different topics percategory. This additional file presents the full output of the topic modeling step from the NLP pipeline. It shows a complete overview of the different topics that were extracted per category.

## Data Availability

The datasets generated and analyzed during the current study are available from the corresponding author (MB) on reasonable request.
