# Supplementary material for: Analyzing patient experiences using natural language processing: development and validation of the artificial intelligence patient reported experience measure (AI-PREM)
Source: BMC Med Inform Decis Mak. 2022 Jul 15;22:183. doi: 10.1186/s12911-022-01923-5 (PMC9284859; doi:10.1186/s12911-022-01923-5)
Supplement: Supplementary file 1 — Additional file 1. Complete overview of the different topics percategory. This additional file presents the full output of the topic modeling step from the NLP pipeline. It shows a complete overview of the different topics that were extracted per category. [file 12911_2022_1923_MOESM1_ESM.pdf]

Additional file 1
Complete overview of the different topics per category

Q1: How was the provided information?

Positive topics	Amount
Good clear, good information, information good, very good, good inform	178
Good clear, information clear, very clear, clear explanation, clear good	178
leftover	3

Negative topics	Amount
Information question, question surgery, bad, so-so, consideration	18
Long ago, testing travel time, day testing, long day, duration	8
leftover	0

Q2: How was the personal approach?

Positive topics	Amount
Very good, very good, good clear, personal approach, good professional	175
Fine alright, good support, also fine, organize well, at ease	55
Very pleasant, very good, very pleasant, good pleasant, pleasant conversation	101
Personal approach, approach excellent, understanding compassionate, compassionate disease	24
leftover	5

Negative topics	Amount
Mutual understanding, could ask, mutual could, matter-of-fact, optimal	3
Personal approach, no personal, vestibular schwannoma practice, scan, contact	3
Quick advise, advise surgery, so-so, pretty quick, very	6
Often present, phone accessible bad, bad accessible, present phone, coordinator then	3
Emotional support, decide emotional, decide treatment, got feeling, treatment take place	4
Very hasty, experience doctor, story good, found, immediately	7
Live life, maybe wave, want normal, wave away, lead think	5
leftover	0

Q3: How was the collaboration between healthcare professionals?

Positive topics	Amount
Collaboration good, went well, all good, good collaboration, very good	215
Collaboration fine, went well, everything fine, gone well, between	98
leftover	12

Negative topics	Amount
Between different, hospital bad, different hospital, bad between, regional hospital	4
Went optimal, situation [city], between [city], [city] went, data exchange	3
Communication between, difficult communication, between hospital, between mental healthcare, so-so name	7
Contact other, other hospital, apparently with, doctor, refer	5
Honor misunderstanding, misunderstanding call, call appointment, good honor, ventilate	3
Make appointment, make scan, question go, sometimes difficult, went sometimes	9
Information surgery, information contact, often, tell story, between themselves	8
leftover	1

Q4: How was the organization of care?

Positive topics	Amount
Organization good, very good, very good, good accessibility, went good	205
Organization fine, fine arrange, fine [hospital], fine alright, appointment	59
Good arrange, fine arrange, perfect arrange, [name] compliment, arrange [name]	62
leftover	17

Negative topics	Amount
Difficult reach phone, bad phone accessibility, so-so bad, case client, bad communication	5
Good, appointment, make, let, well	34
leftover	0

Q5: What else would you like to share about your experience?

Positive topics	Amount
Only good, good experience, experience good, aftercare good, very good	105
Treatment aftercare, only positive, only good, good experience, everyhing fine	8
leftover	8

Negative topics	Amount
Aftercare good, aftercare deal with, deal with new, situation well, new situation	14
Long wait, result scan, wait result, long ago, surgery confess	21
leftover	0
